# A logistic model to predict early pregnancy loss following in vitro fertilization based on 2601 infertility patients

**DOI:** 10.1186/s12958-016-0147-z

**Published:** 2016-03-31

**Authors:** Yan Yi, Guangxiu Lu, Yan Ouyang, Ge lin, Fei Gong, Xihong Li

**Affiliations:** Institute of Reproductive and Stem Cell Engineering, Central South University, Xiangya Road, Changsha, 410078 Hunan P. R. China; Reproductive and Genetic Hospital of CITIC-Xiangya, Xiangya Road, Changsha, 410078 Hunan P. R. China

**Keywords:** Logistic model, Early pregnancy loss, Mean gestational sac diameter, Crown-rump length, Fetal heart rate, In vitro fertilization embryo transfer

## Abstract

**Background:**

According to previous studies, even after embryonic cardiac activity is detected, the pregnancy loss rate remains 3–4 %. The objectives of this study were to investigate the differences in ultrasound parameters between a miscarriage group and an ongoing pregnancy group during the 1^st^ trimester and to build a logistic model to predict early pregnancy loss (EPL) after the appearance of embryonic cardiac activity in patients who have undergone in vitro fertilization embryo transfer (IVF-ET) treatment.

**Method:**

A total of 2601 patients with early singleton pregnancies with embryonic cardiac activity were retrospectively analyzed after IVF from January 2010 to June 2011. Transvaginal sonography (TVS) was performed at 6 to 10 weeks of gestational age (GA). The mean gestational sac diameter (MSD), crown-rump length (CRL), fetal heart rate (FHR), and yolk sac diameter (YSD) were measured by TVS.

**Results:**

A total of 2400 patients had an ongoing pregnancy and an additional 201 (7.7 %) patients miscarried during the first trimester after fetal cardiac activity had been established. The maternal age (MA) and infertility duration were much greater, and the MSD, CRL, and FHR were much lower in the miscarriage group than in the ongoing pregnancy group after IVF (*P* < 0.05). The prediction model utilized the following equation: the possibility of EPL = exp(z)/(1 + exp(z)), where z = −21.456 + (0.114 × MA) + (4.305 × × GA) - (0.043 × MSD) - (0.359 × CRL) - (0.091 × FHR) + 2.243 (fluid collection present around the gestational sac (GS)) + 2.519 (when YSD < 3) or - 0.347 (when YSD > 5.5).

**Conclusion:**

The MA, MSD, CRL, YSD, FHR, infertility duration, and fluid collection around the GS were each correlated with EPL after IVF in infertile patients. A logistic model is a useful tool for predicting EPL after the appearance of embryonic cardiac activity (area under the curve [AUC] = 0.909).

## Background

With the development of transvaginal sonography (TVS), more detailed information may be obtained during early pregnancy [1]. Specifically, the fetal heart rate (FHR) may be measured precisely using M mode TVS [2]. According to previous studies, the spontaneous 1^st^ trimester miscarriage rate is 15–30 % in the general population [3, 4]. Even after embryonic cardiac activity is detected, the pregnancy loss rate remains 3–4 % [5]. Therefore, the development of a prediction model for early pregnancy loss (EPL) is necessary after the appearance of fetal cardiac activity, particularly in infertility patients, who experience tremendous psychological stress and anxiety.

Many parameters can be used to provide a prognosis of pregnancy outcome, and these types of parameters, such as maternal age (MA) and sonographic measurements, are also easily attainable. Fetal bradycardia has been correlated with EPL [6]. One study reported that an FHR less than 90 beats per minute (bpm) is associated with poor pregnancy outcomes [5]. The measurement of the yolk sac size using TVS is also valuable for predicting EPL. An excessively large or small yolk sac, as well as the absence of a yolk sac, is associated with adverse pregnancy outcomes [7, 8]. The ongoing pregnancy rate was much greater in the group in which the yolk sac diameter (YSD) ranged from 2 to 6 mm than that in groups in which the YSD was either less than 2 mm or greater than 6 mm (OR = 33.1, *P* < 0.001) [9]. The mean gestational sac diameter (MSD) and crown-rump length (CRL) are also predictors of EPL [10].

A prediction model including the above four parameters has been established for females who undergo spontaneous conception [2, 11]. When these indicators are used to predict EPL, the gestational age (GA) should also be taken into consideration because the MSD, CRL, YSD, and FHR vary depending on GA. However, for spontaneous conception, the exact GA cannot be determined in females whose last menstrual period was uncertain or whose menstrual cycle is irregular. Thus, in a previous study, the GA was not included in the prediction model, which led to the reduced prediction power of this model. Therefore, the aim of our study was to establish a precise prediction model of EPL in females after IVF-ET treatment because an accurate GA can be determined in this population, increasing the prediction power of the model.

In addition, no large studies have been conducted to verify the relationships between early ultrasound parameters and EPL in infertility patients after IVF. Therefore, our study analyzed the relationship between ultrasound factors and EPL based on data collected from initial TVS scans of 2601 females with viable singleton pregnancies who underwent IVF-ET in our hospital. Using these data, we designed a valuable model to predict EPL, even if embryonic cardiac activity was detected initially.

## Methods

The institutional review board approved this study before data collection. A retrospective chart review was conducted for 4476 infertile patients to identify 2601 patients who had positive fetal cardiac acitivty on their first ultrasound for inclusion into our analysis. All these patients underwent IVF-ET treatment at the Reproductive and Genetic Hospital of CITIC-Xiangya from January 2010 to June 2011.

These 4476 patients underwent controlled ovarian hyperstimulation (using follicle-stimulating hormone or human menopausal gonadotropin), human chorionic gonadotropin (HCG) injection, and blood tests for serum β-HCG 14 days after embryo transfer (ET). A total of 2806 patients had positive results for serum β-HCG. TVS was subsequently performed on these patients to measure the MSD, CRL, YSD, and FHR and to observe fluid collection around the GS from 6 to 10 weeks of GA. The GS was measured in three orthogonal planes, and the data were recorded as the mean gestational diameter. The yolk sac was measured along its longest diameter using a caliper placed along the center wall of the yolk sac [12]. The CRL was measured along the greatest length of the embryo. The FHR was measured using M mode TVS. The ultrasound devices used in this study consisted of either a GE VOLUSON 730 or an E8 (General Electric Tech Co., Ltd., New York, America) instrument housed in the imaging department of our hospital, and all examinations were performed by trained sonographers. A total of 2601 patients exhibited viable (fetal heart activity detected) singleton pregnancies in the initial TVS scan conducted during this study. Multiples were excluded from this study. The MA, infertility duration, sonographic data and early pregnancy outcome of these patients were collected in this study. EPL was defined as miscarriage before or at 12 weeks of gestation and ongoing pregnancy was defined as pregnancy > 12 weeks of gestation.

The differences in the clinical characteristics between the miscarriage group and the ongoing pregnancy group were compared using either a two-sample *t*-test or a Wilcoxon rank sum test. The fertility duration and IVF cycle are expressed as medians [interquartile range] and were compared using the Wilcoxon rank sum test. The MSD, CRL, FHR and YSD in the EPL group and the ongoing pregnancy group were compared during each gestational week using two-sample t-tests and chi-square tests. A logistic regression model was established to assess the odds of pregnancy loss using the ultrasound parameters and additional measurements. A receiver operating characteristic (ROC) curve was obtained to assess the predictive value of this model and the individual ultrasound indicators. The significance level (alpha) was *P* < 0.05 for all analyses. Statistical Package for Social Sciences version 18 (SPSS Inc., Chicago, IL, USA) was used for the data analysis.

## Results

A total of 2400 (92.27 %) patients had an ongoing pregnancy and an additional 201 (7.7 %) patients miscarried during the first trimester after fetal cardiac activity appearance. Both the mean MA (32.67 ± 4.35 vs. 30.59 ± 4.27, *P* < 0.001) and infertility duration (4 [3–7] vs. 5 [3–8.5], *P* = 0.03) were significantly greater in the miscarriage group than in the ongoing pregnancy group. However, the number of IVF treatment cycles was not different between the two groups, and most of the patients in this study underwent only one IVF transfer cycle (1 [1, 2] vs. 1 [1, 2], *P* = 0.93).

**Fig. 1:**
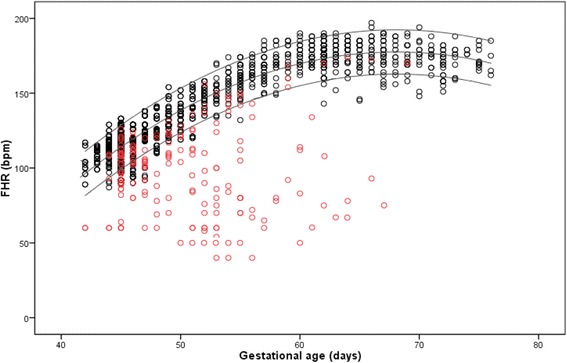
Scatterplot of the changes in fetal heart rate (FHR) for ongoing pregnancy (black circles) and early pregnancy loss (red circles) from 6 to 10 weeks of gestation. The normal range (black line) was derived from the ongoing pregnancy group (median, 95^th^ and 5^th^ percentile)

The MSD, CRL and FHR were compared between the EPL group and the ongoing pregnancy group during each gestational week (Table 1). In the ongoing pregnancy group, the MSD, CRL and FHR were significantly higher than the corresponding values in the EPL group from 6 to 9 weeks of gestation (Table 1, *P* < 0.05). In addition, the FHR increased with GA from 6 to 9 weeks and peaked at approximately 9 weeks (Fig. 1).

**Table 1:** Comparison of the MSD, CRL and FHR in the two groups during each gestational week

Parameters	Ongoing pregnancy	Early pregnancy loss	P	95 % CI
	*n* = 2400	*n* = 201		
MSD (mm)				
6 week	19.95 ± 3.64	17.50 ± 4.82	<0.01	(1.42, 3.47)
7 week	26.42 ± 5.48	19.06 ± 6.68	<0.01	(5.76, 8.97)
8 week	35.70 ± 5.89	24.39 ± 8.2	<0.01	(7.61, 15.02)
9 week	41.16 ± 6.16	30.00 ± 8.94	0.006	(4.26, 18.07)
10 week	48.59 ± 6.27	-	-	-
CRL (mm)				
6 week	3.93 ± 1.134	3.30 ± 1.05	<0.01	(0.383, 0.863)
7 week	9.56 ± 2.34	5.22 ± 2.77	<0.01	(3.672, 5.011)
8 week	18.57 ± 2.58	8.17 ± 5.3	<0.01	(8.030, 12.774)
9 week	24.50 ± 2.57	13.93 ± 11.6	0.026	(1.645, 19.489)
10 week	34.98 ± 3.35	-	-	-
FHR (bpm)				
6 week	115.64 ± 9.25	98.12 ± 17.66	<0.01	(15.421, 19.606)
7 week	144.31 ± 12.36	97.26 ± 32.68	<0.01	(39.665, 54.427)
8 week	172.85 ± 9.12	100.86 ± 40.74	<0.01	(53.887, 90.078)
9 week	175.89 ± 9.27	118.44 ± 50.91	0.010	(18.305, 96.594)
10 week	172.31 ± 9.21	-	-	-

Comparisons were made using a two sample *t*-test

Values are expressed as the means ± standard deviation

*CI* confidence interval

Significant differences were observed in the ongoing pregnancy rate among different YSD ranges (Table 2). The ongoing pregnancy rate was higher (93 %) in the 3–5.5 mm YSD group than in any other YSD group. In the YSD < 3 mm group, the ongoing pregnancy rate was 47.4 %. The miscarriage rate was 21.8 % in patients exhibiting fluid collection around the GS, which was significantly higher than in patients without fluid collection (*P* **<** 0.001).

**Table 2:** Comparison of the YSD and fluid collection around the GS between the two groups

Parameters	Total	Ongoing pregnancy	Early pregnancy loss	P
		n = 2400	n = 201	
YSD (mm)				
<3	19	9 (47.4 %)	10 (52.6 %)	
3-5.5	2420	2251 (93.0 %)	169 (7.0 %)	<0.001
>5.5	162	140 (86.4 %)	22 (13.6 %)	
Fluid collection around GS				
No	2188	2077 (94.9 %)	111 (5.1 %)	<0.001
Yes	413	323 (78.2 %)	90 (21.8 %)	

Comparisons were made using the chi-square test

A logistic regression model was established to assess the effect of the MA, GA, MSD, CRL, FHR, YSD, and fluid collection around the GS (Table 3). Infertility duration was excluded from this model due to stepwise screening. According to this logistic model, the probability of first trimester miscarriage was equal to the following: exp(z)/(1 + exp(z)), where z = −21.456 + (0.114 × MA) + (4.305 × GA) - (0.043 × MSD) - (0.359 × CRL) - (0.091 × FHR) + 2.243 (fluid collection present around the GS) + 2.519 (when YSD < 3) or - 0.347 (when YSD > 5.5).

**Table 3:** Logistic regression analysis of the indicators used to predict EPL

Parameters	β	OR (CI)	P
MA (years)	0.114	1.121 (1.066–1.178)	<0.001
GA (weeks)	4.305	74.088 (32.957–166.551)	
MSD (mm)	−0.043	0.958 (0.910–1.008)	
CRL (mm)	−0.359	0.699 (0.619–0.789)	
FHR (bpm)	−0.091	0.913 (0.898–0.929)	<0.001
YSD (mm)			
3-5.5		1	
<3	2.519	12.421 (3.127–49.343)	<0.001
>5.5	−0.347	0.707 (0.255–1.965)	<0.001
Fluid collection around the GS	2.243	9.422 (6.099–14.558)	<0.001
Constant	−21.456		

The data were analyzed using a binary logistic regression analysis

Method = stepwise (LR). Criteria = PIN (0.05) and POUT (0.10). Model R^2^ = 0.593, *P* < 0.001

Dependent variable assignment: ongoing pregnancy = 0, miscarriage = 1

Independent variable assignment: YSD {mm, 3–5.5 = 1 (Dummy variable), < 3 = 2, > 5.5 = 3}; fluid collection around GS (yes = 1, no = 0)

*OR* odds ratio, *CI* confidence interval

**Fig. 2:**
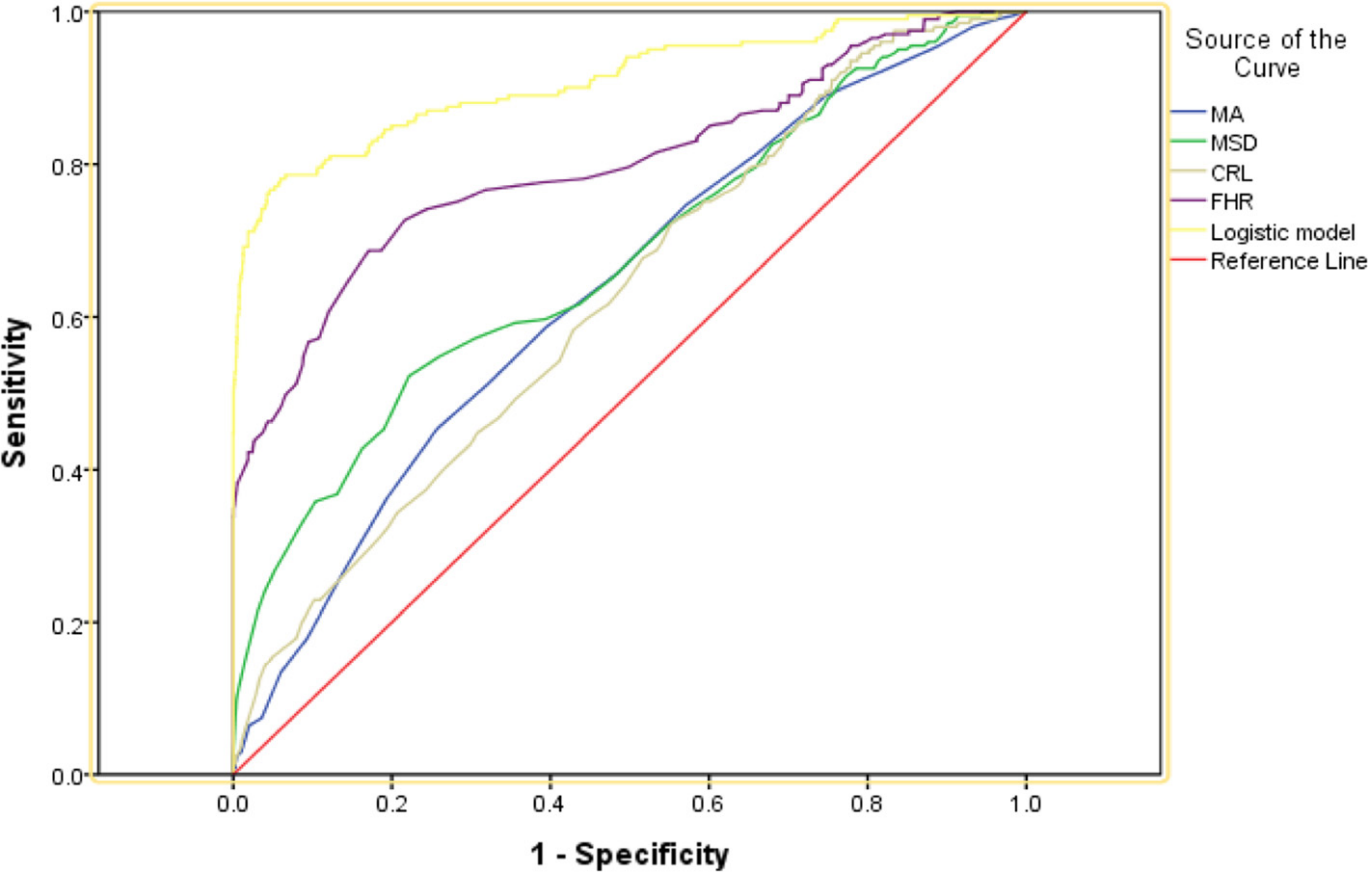
ROC curve analysis for the MA, MSD, CRL, FHR and logistic model for EPL prediction

ROC curve analysis was used to evaluate the predictive value of this logistic model and several individual indicators (MA, MSD, CRL and FHR). Figure 2 depicts the area under the curve (AUC) of the logistic model (AUC = 0.909) and that for the FHR (AUC = 0.800), MSD (AUC = 0.675), MA (AUC = 0.634) and CRL (AUC = 0.623).

## Discussion

Regarding the diagnostic criteria for nonviable pregnancy during the 1^st^ trimester, a previous study used the following guideline for EPL: an MSD ≥ 25 mm and either no embryo or a CRL ≥ 7 mm and no heartbeat [10]. This guideline applies to early pregnancy without fetal cardiac activity. However, for some cases, even after cardiac activity appears initially, there is still a chance that the embryo will be lost. Most of these patients had unfavorable ultrasound parameters such as small GS and CRL. Therefore, it is necessary for clinicians to identify cases that carry a risk of pregnancy failure because repeated TVS is recommended, especially for infertility patients after IVF-ET, in these cases. Therefore, our study established an accurate model to predict EPL after the detection of cardiac activity in infertility patients after IVF. This model includes ultrasound parameters, such as the MSD, CRL, YSD and FHR, which correspond to an accurate GA. According to the ROC curve analysis, the AUC of the logistic model (AUC = 0.909) was greater than that of any other individual parameter.

In this study, a lower FHR was associated with EPL after the use of reproductive assistance technology. A lower FHR may result from an imbalance in the autonomic nervous system [13] and may be correlated with chromosomal anomalies [14]. In trisomy 18, the FHR is decreased; a lower FHR may indicate a pre-terminal event due to severe early-onset growth restriction [15]. Based on the change in FHR in conjunction with the GA in the ongoing pregnancy group (Fig. 1), the FHR peaked at approximately 175.89 bpm at 9 weeks of gestation. However, it is unknown whether parameter is abnormal when the FHR is above the normal reference range at 6–9 weeks of gestation or when the FHR increases with GA even after 9 weeks. The FHR is known to increase in some cases of trisomy 13 at 11–13 weeks of gestation [15]. Trisomy 13 causes left ventricular outflow tract obstruction (LVOTO) [16]; tachycardia may occur as a compensatory mechanism to increase cardiac output [17]. Additional studies focusing on whether fetal tachycardia during the 1^st^ trimester is associated with EPL should be performed.

The yolk sac appearance in the gestational sac is further evidence of an intrauterine pregnancy. Some studies have found no correlation between EPL and either the size or the shape of the yolk sac [18, 19]. These results are inconsistent with the results of our study. The reasons for this inconsistency may include measurement error or a difference in sample size. The yolk sac normally ranges from 2 to 6 mm, with a narrow distribution; therefore, this parameter is difficult to precisely measure. Furthermore, the measurement methods used in different studies were not the same. One study indicated that the yolk sac was measured in three orthogonal planes, from the outer borders of the sac [20]. In another study, the yolk sac was measured from the inner limits of the longest diameter of the yolk sac on a magnified scan [21]. According to our study, the ongoing pregnancy rate was lower in both the larger and smaller YSD groups than in the normal group. The yolk sac plays an important role in the primary exchange between the mother and embryo before the placental circulation is established. In addition to metabolic substance exchange, the yolk sac also has endocrine and hematopoietic functions during early pregnancy [22, 23]. A large yolk sac may be due to yolk sac membrane dysfunction, resulting in collection of secretions. A small yolk sac or no yolk sac may be associated with either dysplasia or early atrophy.

Many studies have reported that advanced MA is associated with poor pregnancy outcomes after IVF [24, 25]. Our study is consistent with this view in that the miscarriage rate increased with age. This result may be attributed to the deterioration of the oocyte and to endocrine variations that occur with aging. The mean MAs in the two groups were significantly different; therefore, this variable was selected for inclusion in the logistic regression analysis.

Fluid collection around the gestational sac may indicate either choriodecidual or amniodecidual separation during early pregnancy, with or without vaginal bleeding, and this is sometimes associated with abdominal pain. This fluid collection presents as an anechoic area around the gestational sac and may be detected easily by TVS. According to our logistic model, fluid collection around the GS is negatively correlated with ongoing pregnancy and is a useful prognostic indicator. Perhaps choriodecidual and amniodecidual separation are indirect signs of the maternal immune system attacking the embryo. Further investigation is needed to determine whether the size, volume, and position of the fluid collection are related to EPL.

The duration of infertility was excluded from this model partly because it is influenced by several confounding factors and partly because it is also a reflection of MA. Therefore, MA is more of a direct factor than the duration of infertility. In addition, number of IVF treatment cycles may not completely reflect the ovarian response because low and high ovarian responses, as in ovarian hyper-stimulation syndrome (OHSS), may both result in transfer cycle cancellation. Therefore, the number of treatment cycles was not included in this model.

One deficiency of this study was that fewer samples were included in the miscarriage group than in the ongoing pregnancy group. Another limitation of this study was that only singleton pregnancies were included. If the multiples were also included in this study, the result analysis will be complicated. First, we need to consider how to record and analyze monochorionic twin or triplet pregnancy (one gestational sac with multiple embryo and yolk sac). Besides, measurements error of ultrasound parameters (MSD, CRL, FHR etc.) will increase in multiple pregnancies sometimes. For example, in a dichorionic twins pregnacy, one of the gestational sac implanted in unfavorable observation position just behind another gestational sac which may lead to incorrect mesurements for ultrasound parameters. Besides, not including whether or not the embryo was biopsied for preimplantation genetic screening (PGS) would also be a limitation for this study. Before applying this model in clinical practice, additional prospective investigations should be performed.

## Conclusion

This study provides a logistic model to predict EPL after the appearance of embryonic cardiac activity in infertility patients. When the predictive results suggest risk of EPL, repeated scans are recommended for these patients, which will help clinicians to determine subsequent interventions.

## Abbreviations

AUC: area under the curve
Bpm: beats per minute
CRL: crown-rump length
EPL: early pregnancy loss
FHR: fetal heart rate
GA: gestational age
GS: gestational sac
IVF-ET: in vitro fertilization embryo transfer
MA: maternal age
MSD: mean gestational sac diameter
OHSS: ovarian hyper-stimulation syndrome
PGS: preimplantation genetic screening
ROC curve: receiver operating characteristic curve
TVS: transvaginal sonography
YSD: yolk sac diameter
